# Extracranial meningioma in the scalp skin 25 years after epidural hematoma surgery: A case report

**DOI:** 10.1016/j.ijscr.2021.105734

**Published:** 2021-03-08

**Authors:** Dinh Hung KIEU, Thị Hien TRINH, Sy Lanh NGUYEN, Hung Manh NGO

**Affiliations:** a Hanoi Medical University, Hanoi, Viet Nam; b Viet Duc Hospital, Hanoi, Viet Nam; c University of Medicine and Pharmacy, Vietnam National University, Hanoi, Viet Nam

**Keywords:** Extracranial meningioma, Neurosurgery, Case report

## Abstract

**Introduction and importance:**

Extracranial meningiomas are rare, accounting for 1–2% of all meningiomas with just published in the form of a case report or series. They are categorized as primary or secondary tumors. In this paper, we present a case of extracranial meningioma in the scalp skin, which is believed in Vietnam's first case.

**Case presentation:**

A 38 years old male with a history of brain epidural hematoma surgery in 1995 presented a subcutaneous protruding mass, which revealed approximately 3-centimeters from an incision scar in the left frontoparietal scalp. The tumor was firm, mobilized with contrast enhancement on magnetic resonance image. The complete surgical excision of the whole mass was performed, and histological examination revealed a WHO grade I meningioma.

**Clinical discussion:**

Epidemiology, classification, diagnosis, treatment as well as prognosis of extracranial meningioma were discussed. We also hypothesized the emergence of extracranial meningioma after an epidural hematoma surgery.

**Conclusion:**

Extracranial meningioma is quite rare, but it could be presented after a traumatic brain injury. Neurosurgery was the first choice, safe and effective treatment.

## Highlights

• Extracranial meningiomas are sporadic, with only published case reports in the literature.
• Extracranial meningiomas are classified into the primary and secondary types, often misdiagnosed before operation due to lack of prevalence and unusual symptoms.
• It is impossible to distinguish between intracranial and extracranial meningiomas regardless of histologic features.
• Surgical resection is the first-choice treatment for extracranial meningiomas.

## 1 Introduction

Meningiomas are the most common benign tumors that account for 20–30% of intracranial tumors [1]. They are a non-glial neoplasm that originates from arachnoid villous structures of meninges. But extracranial meningiomas are rare, constituting 1–2% of all meningioma [2,3]. An applied division of extracranial meningiomas is into primary (rare) and secondary type (which might be seen as a direct extension of an intracranial mass). We herein describe a case that is believed as an unprecedented extracranial meningioma revealed in Vietnam. This case report has been reported in line with the SCARE criteria [4].

## 2 Case presentation

**Fig. 1:**
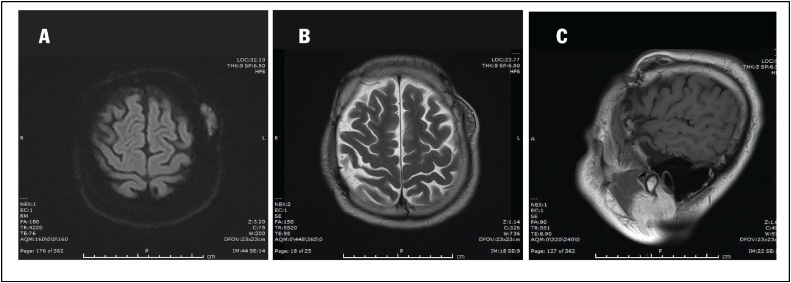
A. Pre-contrast Sagittal T1-weighted sequence demonstrated a well-circumscribed mass separated from the calvarium with isointense to grey matter. B. Pre-contrast axial T1-weighted sequence demonstrated a well-circumscribed mass in the left frontoparietal scalp. C. Pre-contrast axial T2-weighted sequence presented an isointense to grey matter mass.

**Fig. 2:**
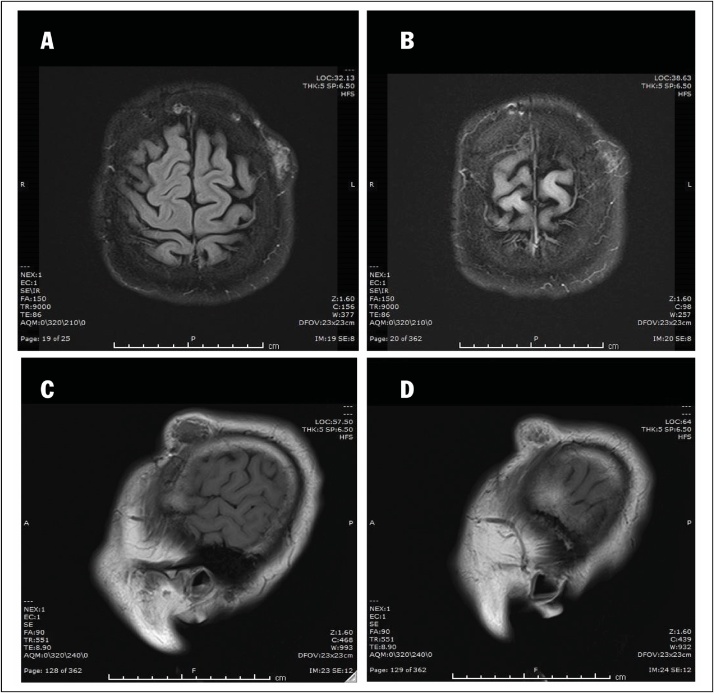
A, B. Post-contrast axial T1-weighted sequence showed a slight homogenous enhancement without any bony invasion. C, D. Post-contrast sagittal T1-weighted sequence showed a homogenous enhance of the left frontoparietal scalp mass, separated from the calvarium.

**Fig. 3:**
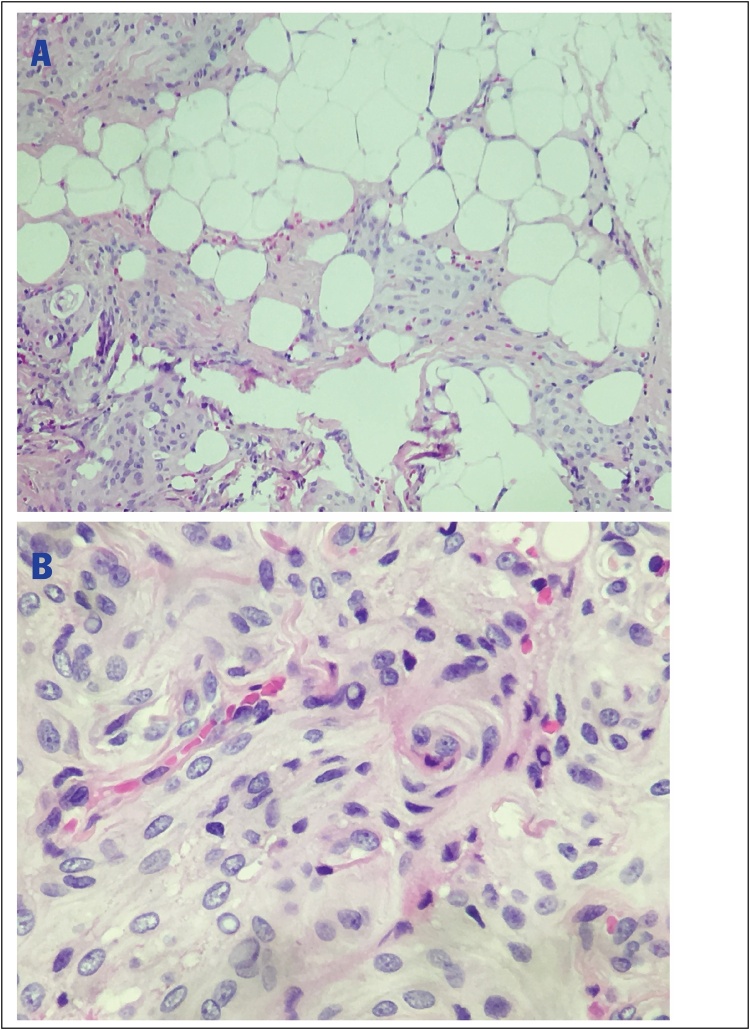
HE × 100 and HE × 400, tumors at the subcutaneous fatty connective tissue (A), the tumor consists of meningoepithelial cells, arranged into the syncytial form of meninges and may appear to be speudoinclusion in the nucleus (B).

**Fig. 4:**
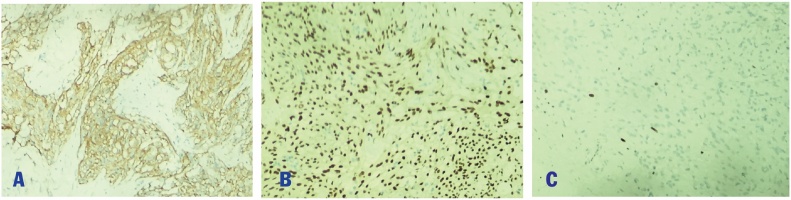
Immunohistochemical staining × 100, tumor cells were positive for EMA (A), positive for PR (B) and low Ki67 rate of about 2% (C).

A 38-year-old man in otherwise good general health presented with a large mass in the left frontoparietal scalp. The mass was progressively enlarged over three years. He gave a history of a prior surgery of bilateral extradural hematoma in 1995. He had an MRI examination in 2018 with the conclusion of lipoma in another medical center. As a result, he was considered to continue observation. Due to the enlargement of the mass, he decided to come to our center. Clinically, it is a firm, mobile, and subcutaneous tumor in the left frontal region, nearly 3 cm from his old scar. He received a preoperative MRI and other routine blood work. The Magnetic resonance imaging (MRI) with Gadolinium revealed a mass in the left frontoparietal scalp, which is well-circumscribed separated from the calvarium (30 mm at the greatest dimension) with slightly low signal intensity on T1-weighted imaging (Fig. 1A) and with some foci of low signal intensity on T2-weighted imaging (Fig. 1B), restricted on DWI without bony invasion. After Gadolinium injection, the mass showed a slight heterogeneous enhancement (Fig. 2A and B). Overall, the conclusion of the radiologist was a benign tumor in the scalp without a specific diagnosis. A scalp skin incision was performed at about 3 cm from postoperative scar under general anesthesia, and the tumor was removed all the involvements entirely as a whole mass. The surgery was made by a senior (Dr. Ngo Manh Hung). Histopathology determined a meningothelial meningioma, which is categorized as grade I according to WHO classification (Fig. 3). The immunohistochemical findings showed positive reactions in the tumor cells for epithelial membrane antigen (EMA) and progesterone receptor (PR). The Ki-67 proliferation index was very low at about 2% (Fig. 4).

## 3 Discussion

Meningioma is a common tumor, occupying 24–35% of the central nervous system's tumors [5,6]. Although they are mostly intracranial, they are sometimes also extracranial tumors that do not have a connection with dura [7]. These are usually referred to as extracranial meningeal tumors or ectopic arachnoid cells, and they only account for 1–2% of general meningioma. Until now, the prior work has only reported individual cases or a series of cases [3,[8], [9], [10], [11], [12]]. Rushing et al. reported 146 cases of primary extracranial tumors during 1970 and 1999, which is considered the most extensive research [7]. It is also interesting to note that even in Vietnamese literature, this topic is also underexplored. This article is the first work that reports an extracranial meningioma found in Vietnam to the best of our knowledge.

Extracranial meningioma could be classified into two forms, which are primary and secondary tumors. Primary tumors are completely extracranial, and not associated with leptomeninges or intracranial mass, while secondary extradural meningiomas refer to intradural meningiomas with extradural extension and/or metastasis [1,13]. The percentage of intracranial meningioma, which has an extradural attachment, was 20%, and orbit is the most prevalent lesion [1,9]. According to this classification, our case could be categorized as a primary extracranial tumor

Primary extracranial tumors are often found in many locations. Rushing at el. analyzed 146 cases of the primary extracranial tumor and reported that common locations, including the skin scalp (n = 59), ear and temporal bone (n = 38), nasal cavity, and nasopharynx (n = 35) [7]. It has also been indicated that the tumors are least found in soft tissue or neck region (n = 14); therefore, they usually appeared in the form of a case report in other studies [8,11,12,14]. Nevertheless, there is no difference in the gender ratio in total Rushing’s cases, but gender imbalance is found in each group/subgroup based on anatomic sites. Specifically, scalp extracranial meningiomas are more prominent in men, while women are more often diagnosed with tumors on the ear and temporal bone sites [7]. These findings are distinctive to intracranial meningiomas, which are slightly frequent in women [6]. The patient's age at presentation for men was younger than women, at 39.6 and 45.6, respectively (p = 0.01). Scalp skin tumors had a younger mean age at present when compared to other anatomic sites (36.2 years compared to ear and temporal bone (50.1 years); sino-nasal tract (47.1 years)). Rushing also claimed that scalp skin location could be helpful to detect could be seen as a contributor to early diagnosis of extracranial meningiomas at earlier stages when they are still small in dimension [7].

Extracranial meningiomas are rarely diagnosed in pre-operation stages because of their infrequency and atypical characteristics, especially when there are no images of intracranial tumors and the association between them [1]. Enhancement of dural mater is not usually visible in the primary extracranial tumors. The pre-op CT-scanner examination is necessary for accessing the bony infiltration or invasion and for the benefit of schedule to craniotomy and removes all involved tumors completely [1]. The differential diagnosis includes the scalp's tumors, which compose the epithelial or mesenchymal cells such as schwannoma, melanoma, paraganglioma, or adnexal tumors, etc.

The first line and most successful outcome treatment for extracranial meningioma are complete or partial surgical excision. Preoperative imaging plays a crucial role in the surgery schedule. Hence, clinicians and radiologists have to discuss all the tumor aspects thoughtfully, the association to other involved lesions, and the degree of bony invasion and underlying dural infiltration. It is essential to expose broadly enough to resection of all complicated extracranial parts [1]. Our case was not intricate in terms of surgical techniques because of its locality and dural separation. We had a surgical intervention consisting of the complete removal of the tumor. It is absorbing that we have acknowledged extracranial meningioma, although we scarcely think about it before.

The treatment strategy of extracranial meningioma is different between primary and secondary types. The primary tumors usually separate without intracranial involvement, so it is uncomplicated to resect completely. In terms of secondary, along the growth and infiltration of tumors exist many problems to accomplish the treatment [1].

Histologically, meningiomas are usually divided into different subtypes; most tumors were meningothelial (77.4%) in the general location and 78% in the scalp. However, the other subtypes of grade I or grade II and III also sometimes present. It is no different regarding histology between intracranial and extracranial tumors [1,7], which Rush et al. mentioned in the ordinary of meningioma. They might arise from several mechanisms, including Arachnoid cells of nerve sheaths emerging from foramina in the skull; (2) Displaced or entrapped Pacchionian bodies from an extracranial location during embryological development; (3) Arachnoid islets displaced by trauma or cerebral hypertension. (4) Undifferentiated mesenchymal cells [7]. Our patient had an epidural hematoma operation; although there is no dural opening, it is possible to leptomeningeal migration through the hole of dural tack up suture to the scalp. By dint of locating around 3-cm from the previous operative scar, the tumor is considered to migrate along the percutaneous epidural drainage path despite inexact evidence.

Rushing et al. revealed that 26 recurrent cases in 110 patients are followed-up for at least ten years [7]. They also determined that the three most essential factors in determining survival are the location in the scalp, meningothelial meningioma histological type, and under 40 years of age [7]. The author also highlighted that the best prognosis is recurrence [7]. The average 10-year survival rate for extracranial meningioma is based on anatomic sites. The highest one is sinonasal tract and scalp skin (98.3% and 93.1%, respectively), while the lowest is ear and temporal bone (85.1%). Still, there were no statistically significant differences (p = 0.736) [7]. Of note, the mean age of cohorts presents in the scalp is lower than that of ear and temporal bone (36.2 compared to 50.1%, respectively). The mean 10-year survival rate also changes by histologic type. The grade I extracranial meningiomas have the best prognosis (91.1%), which is likely considerably higher than 88.9% and 50% for grade II and III, respectively.

## 4 Conclusion

Extracranial meningiomas are relatively rare, but they could be presented after a traumatic brain injury. The anatomic site of involvements is most common at the scalp. Neurosurgery was the first choice, safe and effective treatment.

## Declaration of Competing Interest

The authors report no declarations of interest.

## Funding

The authors declared no funding for this research.

## Ethical approval

The study was approved by the Research Ethics Committee of Hanoi Medical University. The procedures used in this study adhere to the tenets of the Declarations of Helsinki.

## Consent

Written informed consent was obtained from the patient for publication of this case report and accompanying images. A copy of the written consent is available for review by the Editor-in-Chief of this journal on request.

## Author contribution

Hung Dinh KIEU: assessed the protocol, summed up, revised manuscript.

Hien Thi TRINH: followed up, wrote manuscript.

Lanh Sy NGUYEN: data curation, followed up, revised manuscript.

Hung Manh NGO: the main doctor conceived the original idea and operated the patients, wrote and revised manuscript.

## Registration of research studies

Not applicable.

## Guarantor

Hung Manh NGO, MD, PhD.

## Provenance and peer review

Not commissioned, externally peer-reviewed.

## Availability of data and material

Data is available upon reasonable request and with permission of Viet Duc Hospital. No patient or author details are included in the figures.
